# Navigating the Growing Prospects and Growing Pains of Managed Aquifer Recharge

**DOI:** 10.1111/gwat.70029

**Published:** 2025-11-07

**Authors:** Dave Owen, Helen E. Dahlke, Andrew T. Fisher, Ellen Bruno, Michael Kiparsky

**Affiliations:** ^1^ Department of Land, Air and Water Resources University of California Davis CA; ^2^ Department of Earth and Planetary Sciences University of California Santa Cruz CA; ^3^ Department of Agricultural and Resource Economics University of California Berkeley CA; ^4^ Center for Law, Energy & the Environment UC Berkeley School of Law Berkeley CA

## Abstract

Increasing water demands and declining groundwater levels have led to rising interest in managed aquifer recharge. That interest is growing in the United States—the focus of this article—and elsewhere. Increasing interest makes sense; managed aquifer recharge can reduce water‐supply challenges and provide environmental benefits, sometimes with lower costs than alternative water‐management approaches. But managed aquifer recharge also faces growing pains, which will make it difficult for projects to scale up and may limit the benefits provided by those projects that do go forward. Some of the problems arise from the challenges of finding physically suitable locations for managed aquifer recharge; many derive from economics, public policy, and law; and some derive from ways in which managed aquifer recharge could exacerbate traditional equity challenges of water management. But as we explain, there also are potential solutions to these challenges, and the future success of managed aquifer recharge will likely depend on the extent to which these solutions are adopted.

## Introduction

Groundwater recharge is the primary process through which aquifers are replenished. Although recharge is a natural hydrologic process, it can be augmented by human intervention. Managed aquifer recharge (MAR) refers to the purposeful recharge of water to aquifers for subsequent recovery and/or environmental benefit (Dillon et al. 2009). Interest in MAR methods and applications has grown in recent years as groundwater supplies have declined globally (Jasechko and Perrone 2021; Scanlon et al. 2023) and the hydrologic, agricultural, industrial, and municipal systems that depend on those supplies are increasingly threatened by water scarcity. Water managers frequently describe MAR as a promising way to respond to intensifying water crises and an increasingly volatile climate, and to do so without many of the costs and environmental impacts usually associated with storage in surface reservoirs (Taylor 1961). In some places, water managers are relying on MAR to close large gaps between supply and demand, thereby responding to years of chronic groundwater overdraft (Ulibarri et al. 2021; California Department of Water Resources 2023a). Academic interest in MAR has also grown (Department of Water Resources 2018; Dillon et al. 2019).

In this issue paper, we note several common challenges facing MAR projects, and we highlight the need to address those challenges. MAR has been proven effective in many settings. It should receive increasing attention from policymakers and water managers, and it should be applied more often. However, there are reasons why MAR implementation has not kept pace with significant and growing needs, despite broad enthusiasm and limited alternative options. Challenges to MAR, if not addressed, create risks of overestimating the future extent of, and benefits from, MAR, and those overestimations could reduce motivation to pursue other water‐management strategies. Meanwhile, in places where MAR deployment is growing rapidly, other reasons for concern are emerging. Most importantly, absent timely policy action, the greatest beneficiaries of MAR projects may be entities that already hold wealth and power, thereby exacerbating inequality in access to critical water resources.

We argue that practical solutions to these issues are possible, but those solutions will require nuanced awareness and concerted efforts by managers, regulators, and other interested groups. In addition, we identify key policy reforms that could promote effective and equitable application of MAR, for both the short term and into the future. This issue paper focuses on challenges and conditions in the United States, with which we are most familiar, but similar issues are likely to arise elsewhere.

## Exciting Potential, Complicated Reality

MAR introduces surface water to aquifers using a variety of methods. Injection wells and infiltration basins are particularly popular, as is bank filtration through stream and river channels. MAR projects are often motivated by a single primary benefit, which typically is augmenting water supply. However, there is increasing awareness of the potential to design and manage MAR projects to yield multiple benefits, including those often associated with nature‐based solutions (Pecharroman et al. 2021; The Nature Conservancy 2021; Milman et al. 2021b).

As a water‐management strategy, MAR is appealing in many ways (Dillon 2005; Dahlke et al. 2018). Most importantly, it can avoid negative environmental impacts associated with surface reservoirs and can minimize evaporation losses (McCartney 2009). It also can avoid some of the high energy and engineering costs associated with alternative supply‐enhancement options like desalination and wastewater recycling (Nassrullah et al. 2020). By storing surplus water in an aquifer for times of later need, MAR offers a potential mechanism for adapting to climate change (Russo et al. 2013; Meixner et al. 2016). MAR also can turn water flows that otherwise could be problematic, like excess channel flows, stormwater runoff, or wastewater treatment plant discharges, into valuable assets (Kocis and Dahlke 2017). At least in concept, MAR can also help to address a range of other impacts of groundwater mismanagement, including groundwater quality degradation, loss of storage, and impacts to groundwater‐dependent ecosystems. Because of these characteristics, advocacy for and predictions of increased use of MAR are widespread (US EPA 2013; Dillon et al. 2022).

Despite broad appeal, the on‐the‐ground reality often lags aspirations. In some places—Idaho's Snake River Plain, central Arizona, and California's Kern County, for example—MAR has made a significant difference in regional water budgets (Megdal et al. 2014; Kiparsky et al. 2021; Miller et al. 2021a; Milman et al. 2021a). However, these places all still face significant water sustainability challenges, and in many other locations, MAR development has remained limited—both in its geographic reach and in the amounts of water recharged (Dillon et al. 2019).

Two additional problems are emerging. One is MAR overpromising. A clear example comes from California, where state law compels local groundwater managers to produce and implement plans for achieving sustainable groundwater management (California 2025). Many of those sustainability plans rely on undeveloped or aspirational MAR projects to augment existing supplies (Escriva‐Bou et al. n.d.; Ulibarri et al. 2021), and they often do so instead of contemplating stronger restrictions on groundwater extraction. This phenomenon is not new. In multiple western states, problems—and sometimes adverse court decisions—have arisen where development projects counted on inchoate MAR projects as key sources of supply (Court of Appeal 2007; Owen 2021).

The second problem is that in areas where MAR is growing rapidly, that growth risks exacerbating the traditional inequities of water allocation. In the American West, as in many other places, water historically flowed to wealth and power, and water rights and governance systems then reified those traditional patterns of distribution (James 2023; Owen 2024). Much of the recent expansion of MAR involves new appropriations by the old power structures, with water for recharge often benefiting large landowners, or landowner‐controlled water districts, that already have old surface‐water rights (California Department of Water Resources 2023a). Once those landowners have invested money in recharge projects and laid claim to additional water, access to and distribution of excess water for MAR could be much harder to change (Ulibarri et al. 2021; Dillon et al. 2022).

## MAR's Systemic Challenges

Why have these trends emerged? One possibility is that these problems are part of an early stage of broader MAR adoption and operation—temporary “growing pains” during a unique phase of technical and policy advancement. But there are reasons to expect that some of MAR's challenges are systemic, and that these challenges will persist and even grow unless they are addressed on a systemic basis. In this section, we focus on a variety of issues that pose challenges for MAR. Not all these challenges are unique to MAR projects, but all are likely to be particularly salient for MAR.

### Site Suitability

For several reasons, it can be challenging to find physically and biogeochemically suitable sites for MAR (O'Geen et al. 2015; Russo et al. 2015; Levintal et al. 2023). First, water of sufficient quality and quantity must be available to supply a MAR project of the scale being planned (McCurry and Pyne 2022). Second, there must be an appropriate means for conveying water from its source to its storage location in the receiving aquifer. Third, hydrogeologic properties of the target aquifer(s) must support source‐water infiltration or injection and provide sufficient transmission and storage space (Bouwer 2002; de Vries and Simmers 2002). Fourth, the chemical compatibility and treatment needs of both the source water and groundwater may need to be considered, accounting for the nature of soils and aquifer materials, as introduced or mobilized contaminants can impact long‐term operational maintenance and water treatment needs and human and aquatic system health (Fakhreddine et al. 2020, 2021; Alam et al. 2021). The possibility of health impacts is particularly important if the project will be located close to existing water‐supply wells (Asano and Cotruvo 2004; Marwaha et al. 2021).

### Economics

Developing MAR projects that are economically viable can also be challenging. MAR is likely to be most economically valuable in places where water is scarce and in considerable demand. But in those same places, there usually is limited water available for recharge, or acquiring that water can be prohibitively expensive, either because that development would require using expensive technologies or because owners of water rights are reluctant or even unable to sell water (Green‐Nylen et al. 2017). These difficulties may be manageable if water is intermittently abundant (as it is, e.g., in the American Southwest), but intermittent availability may mean that MAR systems operate infrequently and/or unpredictably, which can make it hard for the project to generate enough benefit to offset its capital and operating costs (Vanderzalm et al. 2022). And while an assessment of local and regional climate can help project developers assess the potential availability of excess water for MAR over time, flow predictions are often highly uncertain.

Second, the costs and benefits of MAR projects can be difficult to assess. This is partly because of the likelihood of unpredictable and intermittent operation (Bruce et al. 2023), but several other factors can complicate economic assessment (Ross and Hasnain 2018a). Permitting and engineering costs can be difficult to predict, especially where experience designing and permitting MAR projects is limited. Studies analyzing the costs from past projects suggest that the range of costs is broad and that the magnitude of costs depends on numerous site‐specific factors, including the infiltration method, frequency of use, and whether secondary treatment is needed (Ross and Hasnain 2018b). On the benefits side, it can be challenging to place a value on additional water because that value is often dynamic, context‐specific, and difficult to observe (D'Odorico et al. 2020). For a good that is traded in a competitive market, the market price serves as a proxy for its marginal value. Water, in contrast, is often extracted and distributed through systems in which users pay only for extraction and delivery costs and not for the water itself (Ward and Michelsen 2002; Hanemann 2005). Additional potential benefits, such as ecosystem services, are notoriously difficult to value in unambiguous ways and are often ignored (Griebler and Avramov 2015). Risk‐averse water managers may be reluctant to invest in MAR when costs and benefits are hard to characterize with confidence.

Another layer of challenges can arise if MAR projects (along with other supply‐side management activities in a basin) begin to succeed. Improvements that raise groundwater levels could lead to lowered pumping costs, which could encourage more resource use (Koundouri 2004; Olmstead and Stavins 2009; Smith et al. 2017; Bruno and Jessoe 2021). Success in groundwater management could also impact attitudes toward pumping; it is harder to motivate people to conserve a resource that seems increasingly abundant (Berbel and Esteban 2019). For both reasons, MAR projects could eventually become partial victims of their own success. That is not a reason to avoid such projects, but it counsels caution against overestimating the benefits MAR projects will provide.

### Legal Frameworks

Some of the largest challenges facing MAR are legal (Doremus and Job 2022). For a MAR project to succeed, project operators will typically need legal access to source water—which, in the United States, generally means obtaining water rights—regulatory approvals for project infrastructure and operations, and some assurance that they will be able to retain some of the benefits of their investments for themselves—among other likely requirements (Ulibarri et al. 2021). A well‐designed legal regime also would include protections against misuse of MAR projects. For example, water introduced into the subsurface may take months or even years to reach an aquifer, with some potential loss along the way, and recharged water tends to migrate and can leave the region of intended storage (Flint et al. 2000; Bekele et al. 2014). These hydrologic complexities necessitate rules that preclude MAR project operators from pumping too much water or pumping that water too soon—or from claiming too much credit if the project is designed to provide benefits other than supporting water extraction. To be effective, those rules will need to be accompanied by effective monitoring and accounting systems; enforcing rules is difficult, at best, where information about pumping and water levels is absent (Ostrom 2015; Peterson et al. 2024). Finally, a strong legal regime might take steps to ensure that MAR projects provide multiple benefits and provide for an equitable distribution of those benefits. Successful MAR, in short, will typically require robust law.

In many places, however, those basic legal elements do not exist. Some states within the United States have dedicated permitting processes for MAR projects (California State Water Resources Control Board 2025), but most do not. In the latter states, MAR‐project permitting either happens under other laws, like the Safe Drinking Water Act (US EPA 2015), that only apply to some types of projects and only to some project impacts, or there may be no clear permitting pathway at all. Similarly, rules governing MAR‐project accounting are uneven. Most importantly, many states have lax or spotty regulatory regimes for general oversight of groundwater use. Even states that purport to have strong groundwater‐use regulations often carve out significant regulatory exemptions and have major gaps in monitoring and enforcement, with important groundwater uses—like agriculture—largely exempted from legal oversight (Owen 2013; Searcey and Erdenesanaa 2023). Adding to the challenges, in some places, like parts of Texas and Arizona, MAR projects must operate within legal regimes that purport to allow unfettered pumping rights, potentially insulating traditional groundwater users from regulatory control (Tadych et al. 2024). Consequently, in many places, MAR‐project operators have no legal guarantee that water they put into the ground will be available to serve its planned purposes (Bray 2020). It could just be pumped by someone else.

While these legal challenges are particularly salient in the United States, they also are likely to arise elsewhere. US water law is distinctive in some ways, including its emphasis, in some places, on private rights and markets and in its degree of state‐to‐state variation (Adkins et al. 2022). Nevertheless, reluctance to monitor and regulate groundwater use is a recurring theme across a variety of legal systems (Karandish et al. 2025).

### Overpromising

Even as a combination of physical, economic, and legal issues makes many MAR projects difficult to pursue, overpromising is likely to persist. The reason is straightforward: in many situations, the alternatives to pursuing MAR projects are unpopular or even painful (California Department of Water Resources 2023b). Indeed, part of what makes MAR so attractive is that it appears to offer the possibility of lowering or even eliminating the need to restrict groundwater extractions. Those restrictions could reduce agricultural productivity, limit industrial activity, and impact municipal water supply, each of which can harm regional economies and create opposition from water users (Hanak et al. 2023). For government officials, that means the alternative to placing faith in groundwater recharge programs will often be an extra dose of political pain. One does not need complicated theories of behavioral psychology to know that many people will try to avoid these situations—or that they will use some combination of strategic behavior and wishful thinking to try to avoid undesired restrictions on pumping (Thompson 2000).

The challenges of MAR implementation are also likely to result in inconsistencies between projected and actual MAR‐project benefits. The public and some water managers may assume that adding MAR projects will result in less oversight of other water uses. MAR, in other words, might seem to promise an alternative to heightened regulatory attention. But MAR projects are unlikely to succeed, or to demonstrate their value, without careful monitoring of recharge, aquifer storage, and groundwater extractions, which means MAR implementation will often need to be coupled with greater requirements for monitoring and reporting (Casanova et al. 2016; Ulibarri et al. 2021; Doremus and Job 2022; Peterson et al. 2024). Those requirements may be seen by many groundwater users as intrusive and/or expensive, which in turn could erode enthusiasm for MAR projects, even in places where the assumption that those MAR projects would proceed once was the basis for reduced restrictions on pumping. Some of these frustrations may diminish over time, but especially as new projects are developed, enhanced monitoring and reporting could undermine support for MAR (Yuan et al. 2016; Zheng et al. 2023; Sufyan et al. 2024).

### Equity

MAR's emerging equity challenges likewise have structural explanations, and those explanations are rooted in a mixture of economics, law, and history. Pursuing a MAR project often requires water rights, along with sufficient capital to fund project infrastructure, operations, and maintenance. Large landholdings also may be necessary. Some common recharge techniques, like infiltration basins or flooding of working lands or floodplains, need ample space (Meles et al. 2024), and in areas where legal protections against third‐party pumpers are lacking, a large land area may be necessary to ensure that no one else pumps the water. Finally, pursuing a project may require access to expertise, particularly if building and running the project raise novel challenges, require understanding of complex subsurface conditions, and/or involve navigating complex and ambiguous regulatory regimes.

In general, only relatively well‐funded entities can meet these requirements. By definition, lower‐income communities lack access to economic and social capital and to large cash flows, and they generally do not have large landholdings or access to experienced consultants and attorneys. Nor do many lower‐income communities have large or senior surface water rights, as those rights were mostly claimed by people who had wealth and power in past decades and centuries, and many of those power structures persist today (Owen 2024). There are exceptions to these generalizations. Some lower‐ or middle‐income people receive their water from reasonably well‐funded urban water suppliers, and those water suppliers have their own water rights and, sometimes, substantial landholdings (Owen 2021; San Francisco Water Power Sewer 2025). But urban water rights are often more junior than those held by agricultural water users (Tarlock 2001), and cities' water‐supply landholdings may be in more upland areas, which may be good for supplying high‐quality surface water but not for storing groundwater.

MAR could exacerbate these inequities. In the western United States, and increasingly also in the East, water law favors first actors; would‐be new users often must show that they will not interfere with the water rights of entities that have already obtained rights (Thompson et al. 2025). That means that if traditionally powerful users are the first entities to establish MAR projects and claim additional flows, then MAR will become a mechanism for those entities to increase their control of water systems. That increased control could simply mean that lower‐income communities do not partake in the benefits of MAR systems, but it also could mean more direct detrimental effects. Water allocation sometimes is a zero‐sum contest, and if relatively wealthy and powerful actors can use MAR to claim more water, others may wind up with less.

## Helping MAR Succeed

The challenges we have described are significant and may sometimes be unavoidable. The physical challenges of site selection, for example, will always be present, as will the economic challenge of MAR projects being needed most in places where surplus water for use in MAR projects is scarce. Yet even unavoidable challenges can be ameliorated or mitigated, and other challenges are resolvable. Most importantly, providing more guidance on MAR can help project designers, legal frameworks for MAR can develop and change, and MAR projects can be structured in ways that produce multiple public benefits.

### Information

Saying that more information will help MAR projects succeed may sound obvious, but it remains important and true. At present, proponents of MAR projects have an increasing number of case studies to draw upon (UNESCO 2021; Miller et al. 2021b), but more data are needed to cover the geographical, technical, and institutional variations that project proponents are likely to encounter. Creating standardized formats for reporting on MAR projects and professional incentives for doing so could help build a broader information base and make this information more widely available. Improving that information base can help all project designers, and it may be particularly helpful for those with less access to expensive expertise. Similarly, more systematic studies of MAR economics and of regulatory regimes would provide valuable guidance for future projects.

### Regulating Groundwater Extractions

Among potential policy reforms, the most important is to strengthen regulatory regimes for traditional groundwater pumping. MAR will be, at most, a limited substitute for curbing extraction in overpumped basins; there probably are few, if any, overpumped basins where MAR alone can stabilize groundwater levels, never mind making up for decades of overdraft (Liu et al. 2022). California, for example, has become a hotbed of MAR activity, yet experts agree that MAR can help to resolve only part of the state's groundwater‐management challenges (Bland 2023; Hanak et al. 2025). MAR also will be more appealing and will function better where pumping restrictions are in place. People are unlikely to invest in MAR if overpumping an aquifer remains an attractive short‐term option (Texas House Research Organization 2000; Ali et al. 2023). And running a MAR project—particularly a large project or program—will generally be easier if other groundwater users' actions are carefully monitored and are subject to clear and strict regulatory controls, coupled with transparent reporting and accountability (Peterson et al. 2024). In short, MAR will likely be most effective when implemented along with strong regulation of groundwater pumping and its impacts; MAR is not an alternative to such regulation (Peterson et al. 2024).

Importantly, regulatory regimes for groundwater extraction can be structured to provide direct incentives for MAR. In some settings, water regulators have encouraged MAR by creating default regulatory obligations and then allowing MAR‐project operators to obtain flexibility or relief from those obligations. For example, in California's Pajaro Valley, landowners implementing specific kinds of MAR projects can obtain reductions in their groundwater‐pumping fees. These reductions motivate MAR‐project implementation and offset operations and maintenance costs (Bruce et al. 2023). Similarly, municipal stormwater utilities often give fee reductions to landowners who take steps to increase on‐site water infiltration (National Association of Flood and Stormwater Management Agencies 2006). In Virginia's coastal plain, a somewhat similar model exists; wastewater treatment plants' participation in a MAR project helps them comply with Clean Water Act restrictions on nutrient discharges while also helping address a regional problem with groundwater overdraft (Nylen 2021). In other places—California's Kaweah Groundwater Basin, for example—pursuing a MAR project can create flexibility in an otherwise rigid cap on pumping (Mid‐Kaweah GSA 2024). The shared idea is that where regulatory obligations exist as a default and MAR offers a path to compliance, MAR is more likely to succeed.

These regulatory structures offer another significant advantage: they make it easier to allocate the recharged water to collective benefits. In situations where MAR projects are not incentivized by regulatory relief, a private banking model is likely to emerge (Kiparsky et al. 2021). People (or government or corporate entities) are likely to put water in the ground with the intent of taking that water back out. In a regulatory‐relief model, in contrast, some public entity could manage the recharged water; the management model would be like an in‐lieu fee program for compensatory mitigation for wetland impacts (Kihslinger et al. 2019). That public entity may have more flexibility to devote that water to actions with broader public benefits, like avoiding subsidence, bolstering water supplies for lower‐income communities, or promoting environmental flows.

### Public MAR

Water managers should consider pursuing more public MAR projects and programs. If MAR projects are left primarily to private entities, or to public entities that are largely subject to private control (Kiparsky et al. 2021; Owen 2021), then MAR is likely to become a mechanism for consolidating the traditional power structures of water management, and opportunities for public benefit will be lost. We do not suggest that private MAR projects all lack public benefits; they may still help with regional overdraft problems and with sustaining agricultural economies, both of which will benefit people beyond the direct funders of the MAR program. Private benefits also are appropriate motivations for projects, particularly where the project will not create costs for the broader public. But the intentional pursuit of broader public benefits may be easier if public‐serving entities sometimes play a central role in selecting, developing, and operating MAR projects. Those entities then will be positioned to ensure that some project benefits go to things like supporting lower‐income communities or providing environmental protection, rather than attempting to convince private entities to provide those benefits.

### Allocation for the Public Good

Finally, and especially where MAR projects operate for private benefit, MAR regulation should require allocation of water to the affected aquifer and/or to public purposes. These allocations will accomplish two main goals. One goal is to ensure that MAR project operators do not extract too much water. Not all infiltrated water reaches an aquifer, not all groundwater remains in an aquifer, not all water in an aquifer is recoverable, and water that does eventually reach an aquifer and is recoverable may not be recoverable for a long time (Kourakos et al. 2019; Barfield 2021; Levintal et al. 2023). Project operators should be required to leave enough recharged water in an aquifer to compensate for these losses and delays.

Allocating water to an aquifer also can be an important mechanism for ensuring public benefits. Leaving recharged water in an aquifer might help to raise regional groundwater levels, thereby avoiding future subsidence, or could contribute to environmental flows of surface water. Some portion of stored water also might be dedicated to supporting nearby disadvantaged communities or to providing health and safety supplies during future droughts. MAR‐project operators may resist these kinds of allocations, but the dedication of some water is a reasonable expectation. Project operators are using state‐owned resources—water, in every U.S. state, remains partly public property, even when put to private use—and will often be using them for private gain (Sax 1990; Ayres 2022). Providing some public benefit is a reasonable expectation in exchange for that privilege.

## Conclusion

Expanded implementation of MAR is a likely and potentially valuable response to growing water crises. As important as this expansion will be, it carries risks, and closing the gaps between MAR's seeming promise and its on‐the‐ground reality will be challenging. Academics, federal, state, and local decision makers, NGOs, and affected communities each have roles to play. A list of all those roles would be the subject of another article—or book—but a few tasks will be particularly important for addressing the challenges identified in this article. For academics, key tasks will include generating ideas and tools for understanding project siting, performing economic analyses, and building monitoring frameworks, as well as continuing to prepare synthesis studies of existing MAR projects and to educate future water managers, technicians, economists, and lawyers. For state and local decision‐makers, building more robust frameworks and financial incentives for MAR permitting and accounting will be key, as will the thankless but crucially important work of building stronger regulatory frameworks for groundwater extractions. Also important will be efforts to encourage the development of MAR projects that provide multiple benefits. In the United States, where groundwater management remains primarily a state‐ and local‐government responsibility (Carter et al. 2020), the federal government will nevertheless continue to have an important role in providing funding and technical support. And affected communities will need to hold decision‐makers accountable.

If these roles are played well, MAR can be an important, if partial, component of more effective and equitable water‐management systems.

## Data Availability

The article is an issue paper, and it does not draw directly on an original data set.

## References

[gwat70029-bib-0001] Adkins, A. , J. Beasley , R. Bratcher , J. Cias , J. Field , I. Gaunt , A. Graves , M. Hayashi , J. Lusk , M. Maslanka , E. Milliken , C. Pabich , M. Reed , A.W. Remschel , L. Thomas , and A. Wilde . 2022. Groundwater Laws and Regulations: Survey of Sixteen U.S. States. EENRS Program Reports & Publications 2, 1–357. 10.37419/EENRS.USStateGroundwaterLaws.2022

[gwat70029-bib-0002] Alam, S. , A. Borthakur , S. Ravi , M. Gebremichael , and S.K. Mohanty . 2021. Managed aquifer recharge implementation criteria to achieve water sustainability. Science of the Total Environment 768: 144992. 10.1016/j.scitotenv.2021.144992 33736333

[gwat70029-bib-0003] Ali, A.A. , D.Q. Tran , K.F. Kovacs , and H.E. Dahlke . 2023. Hydro‐economic modeling of managed aquifer recharge in the lower Mississippi. JAWRA Journal of the American Water Resources Association 59: 1413–1434. 10.1111/1752-1688.13141

[gwat70029-bib-0004] Asano, T. , and J.A. Cotruvo . 2004. Groundwater recharge with reclaimed municipal wastewater: health and regulatory considerations. Water Research 38: 1941–1951. 10.1016/j.watres.2004.01.023 15087175

[gwat70029-bib-0005] Ayres, S.T. 2022. State water ownership and the future of groundwater management. The Yale Law Journal 131: 2020–2386.

[gwat70029-bib-0006] Barfield, J. 2021. Multi‐Benefit Recharge Project Methodology for Inclusion in Groundwater Sustainability Plans. The Nature Conservancy.

[gwat70029-bib-0007] Bekele, E. , B. Patterson , S. Toze , A. Furness , S. Higginson , and M. Shackleton . 2014. Aquifer residence times for recycled water estimated using chemical tracers and the propagation of temperature signals at a managed aquifer recharge site in Australia. Hydrogeology Journal 22: 1383–1401. 10.1007/s10040-014-1142-0

[gwat70029-bib-0008] Berbel, J. , and E. Esteban . 2019. Droughts as a catalyst for water policy change. Analysis of Spain, Australia (MDB), and California. Global Environmental Change 58: 101969. 10.1016/j.gloenvcha.2019.101969

[gwat70029-bib-0009] Bland, A. 2023. Ground Zero: Rain Brings Little Relief to California's Depleted Groundwater CalMatters

[gwat70029-bib-0010] Bouwer, H. 2002. Artificial recharge of groundwater: hydrogeology and engineering. Hydrogeology Journal 10: 121–142. 10.1007/s10040-001-0182-4

[gwat70029-bib-0011] Bray, Z. 2020. The fragile future of aquifer storage and recovery. San Diego Law Review 57: 1–59.

[gwat70029-bib-0012] Bruce, M. , L. Sherman , E. Bruno , A.T. Fisher , and M. Kiparsky . 2023. Recharge net metering (ReNeM) is a novel, cost‐effective management strategy to incentivize groundwater recharge. Nature Water 1: 855–863. 10.1038/s44221-023-00141-1 PMC13533828 42688042

[gwat70029-bib-0013] Bruno, E.M. , and K. Jessoe . 2021. Missing markets: Evidence on agricultural groundwater demand from volumetric pricing. Journal of Public Economics 196: 104374. 10.1016/j.jpubeco.2021.104374

[gwat70029-bib-0014] California Department of Water Resources . 2023a. California Water Plan Update 2023

[gwat70029-bib-0015] California Department of Water Resources . 2023b. Groundwater Demand Management, in: California Groundwater Projects Tool Project. p. 33.

[gwat70029-bib-0016] California Department of Water Resources . 2025. Sustainable Groundwater Management Act (SGMA). https://water.ca.gov/Programs/Groundwater‐Management/SGMA‐Groundwater‐Management (accessed August 6, 2025).

[gwat70029-bib-0017] California State Water Resources Control Board . 2025. Temporary Permits for Groundwater Recharge. https://www.waterboards.ca.gov/waterrights/water_issues/programs/applications/groundwater_recharge/temporary_permits.html (accessed October 24, 2025).

[gwat70029-bib-0018] Carter, N.T. , P. Folger , C.V. Stern , and M. Stubbs . 2020. The Federal Role in Groundwater Supply (legislation No. R45259).

[gwat70029-bib-0019] Casanova, J. , N. Devau , and M. Pettenati . 2016. Managed aquifer recharge: An overview of issues and options. In Integrated Groundwater Management: Concepts, Approaches and Challenges. 413–434. Springer Nature. https://link.springer.com/book/10.1007/978‐3‐319‐23576‐9.

[gwat70029-bib-0020] Court of Appeal, 3rd District of California . 2007. Vineyard Area Citizens for Responsible Growth, Inc. v. City of Rancho Cordova.

[gwat70029-bib-0021] Dahlke, H.E. , G.T. LaHue , M.R.L. Mautner , N.P. Murphy , N.K. Patterson , H. Waterhouse , F. Yang , and L. Foglia . 2018. Managed Aquifer Recharge as a Tool to Enhance Sustainable Groundwater Management in California. Advances in Chemical Pollution, Environmental Management and Protectio 3: 215–275. 10.1016/bs.apmp.2018.07.003.

[gwat70029-bib-0022] Department of Water Resources . 2018. Flood‐MAR – Using Flood Water for Managed Aquifer Recharge to Support Sustainable Water Resources.

[gwat70029-bib-0023] Dillon, P. 2005. Future management of aquifer recharge. Hydrogeology Journal 13: 313–316. 10.1007/s10040-004-0413-6

[gwat70029-bib-0024] Dillon, P. , A. Kumar , R. Kookana , R. Leijs , D. Reed , S. Parsons , and G. Ingleton . 2009. Managed Aquifer Recharge – Risks to Groundwater Dependent Ecosystems – A Review.

[gwat70029-bib-0025] Dillon, P. , P. Stuyfzand , T. Grischek , M. Lluria , R.D.G. Pyne , R.C. Jain , J. Bear , J. Schwarz , W. Wang , E. Fernandez , C. Stefan , M. Pettenati , J. van der Gun , C. Sprenger , G. Massmann , B.R. Scanlon , J. Xanke , P. Jokela , Y. Zheng , R. Rossetto , M. Shamrukh , P. Pavelic , E. Murray , A. Ross , J.P. Bonilla Valverde , A. Palma Nava , N. Ansems , K. Posavec , K. Ha , R. Martin , and M. Sapiano . 2019. Sixty years of global progress in managed aquifer recharge. Hydrogeology Journal 27: 1–30. 10.1007/s10040-018-1841-z

[gwat70029-bib-0027] Dillon, P.J. , A. Allen , A. William , Y. Zheng , and J. Vanderzalm . 2022. Managed Aquifer Recharge: Overview and Governance, IAH Special Publication. International Association of Hydrogeologists. https://recharge.iah.org/files/2022/06/MAR‐overview‐and‐governance‐IAH‐Special‐Publication‐18June2022.pdf.

[gwat70029-bib-0028] D'Odorico, P. , D.D. Chiarelli , L. Rosa , A. Bini , D. Zilberman , and M.C. Rulli . 2020. The global value of water in agriculture. PNAS 117: 21985–21993. 10.1073/pnas.2005835117 32839335 PMC7486753

[gwat70029-bib-0029] Doremus, L. , and C. Job . 2022. Key elements of a state regulatory framework for managed aquifer recharge. Groundwater 60: 591–601. 10.1111/gwat.13242 35947037

[gwat70029-bib-0030] Escriva‐Bou, A. , E. Hanak , S. Cole , and J. Medellín‐Azuara . n.d. The Future of Agriculture in the San Joaquin Valley, Technical Appendix.

[gwat70029-bib-0031] Fakhreddine, S. , B.R. Scanlon , J.P. Nicot , and M. Young 2020 Assessing groundwater quality impacts of capturing high magnitude flows for managed aquifer recharge 2020, H043‐08.

[gwat70029-bib-0032] Fakhreddine, S. , B.R. Scanlon , and J.‐P. Nicot . 2021. Guidance for Understanding and Minimizing the Potential for Arsenic Mobilization during Aquifer Storage and Recovery (No. TCEQ AS‐218). University of Texas at Austin.

[gwat70029-bib-0033] Flint, A.L. , L.E. Flint , J.A. Hevesi , F. D'Agnese , and C. Faunt . 2000. Estimation of regional recharge and travel time through the unsaturated zone in arid climates. In Dynamics of Fluids in Fractured Rock, 115–128. American Geophysical Union (AGU). 10.1029/GM122p0115

[gwat70029-bib-0034] Green‐Nylen, N. , M. Kiparsky , K. Archer , K. Schnier , and H. Doremus . 2017. Trading Sustainably. Wheeler Water Institute|Center for Law, Energy & the Environment UC Berkeley School of Law.

[gwat70029-bib-0035] Griebler, C. , and M. Avramov . 2015. Groundwater ecosystem services: a review. Freshwater Science 34: 355–367. 10.1086/679903

[gwat70029-bib-0036] Hanak, E. , A. Ayres , C. Peterson , A. Escriva‐Bou , S. Cole , and Z.J. Morales . 2023. Managing Water and Farmland Transitions in the San Joaquin Valley. Public Policy Institute of California. https://www.ppic.org/publication/managing‐water‐and‐farmland‐transitions‐in‐the‐san‐joaquin‐valley/ (accessed October 24, 2025).

[gwat70029-bib-0037] Hanak, E. , S. Cole , G. Cartrell , and C. Peterson . 2025. How Much Water Is Available for Groundwater Recharge in the Central Valley? Public Policy Institute of California.

[gwat70029-bib-0038] Hanemann, W.M. 2005. The economic conception of water.

[gwat70029-bib-0039] James, I. 2023. “A foundation of racism”: California's antiquated water rights system faces new scrutiny. Los Angeles Times. https://www.latimes.com/environment/story/2023‐03‐06/is‐californias‐antiquated‐water‐rights‐system‐racist (accessed February 12, 2025).

[gwat70029-bib-0040] Jasechko, S. , and D. Perrone . 2021. Global groundwater wells at risk of running dry. Science 372: 418–421. 10.1126/science.abc2755 33888642

[gwat70029-bib-0041] Karandish, F. , S. Liu , and I. de Graaf . 2025. Global groundwater sustainability: A critical review of strategies and future pathways. Journal of Hydrology 657: 133060. 10.1016/j.jhydrol.2025.133060

[gwat70029-bib-0042] Kihslinger, R. , C. Libre , K. Ma , E. Okuno , and R.C. Gardner . 2019. In‐lieu fee mitigation: Review of program instruments and implementation across the country. SSRN Journal. 10.2139/ssrn.3619484

[gwat70029-bib-0043] Kiparsky, M. , K. Miller , P. Goulden , A. Milman , and D. Owen . 2021. Groundwater recharge for a regional water bank: Kern water Bank, Kern County, California. Case Studies in the Environment 5: 1223400. 10.1525/cse.2021.1223400

[gwat70029-bib-0044] Kocis, T.N. , and H.E. Dahlke . 2017. Availability of high‐magnitude streamflow for groundwater banking in the Central Valley, California. Environmental Research Letters 12: 084009. 10.1088/1748-9326/aa7b1b

[gwat70029-bib-0045] Koundouri, P. 2004. Current issues in the economics of groundwater resource management. Journal of Economic Surveys 18: 703–740. 10.1111/j.1467-6419.2004.00234.x

[gwat70029-bib-0046] Kourakos, G. , H.E. Dahlke , and T. Harter . 2019. Increasing groundwater availability and seasonal base flow through agricultural managed aquifer recharge in an irrigated basin. Water Resources Research 55: 7464–7492. 10.1029/2018WR024019

[gwat70029-bib-0047] Levintal, E. , M.L. Kniffin , Y. Ganot , N. Marwaha , N.P. Murphy , and H.E. Dahlke . 2023. Agricultural managed aquifer recharge (Ag‐MAR)—A method for sustainable groundwater management: A review. Critical Reviews in Environmental Science and Technology 53: 291–314. 10.1080/10643389.2022.2050160

[gwat70029-bib-0048] Liu, P.‐W. , J.S. Famiglietti , A.J. Purdy , K.H. Adams , A.L. McEvoy , J.T. Reager , R. Bindlish , D.N. Wiese , C.H. David , and M. Rodell . 2022. Groundwater depletion in California's Central Valley accelerates during megadrought. Nature Communications 13: 7825. 10.1038/s41467-022-35582-x PMC9763392 36535940

[gwat70029-bib-0049] Marwaha, N. , G. Kourakos , E. Levintal , and H.E. Dahlke . 2021. Identifying agricultural managed aquifer recharge locations to benefit drinking water supply in rural communities. Water Resources Research 57: e2020WR028811. 10.1029/2020WR028811

[gwat70029-bib-0050] McCartney, M. 2009. Living with dams: managing the environmental impacts. Water Policy 11: 121–139. 10.2166/wp.2009.108

[gwat70029-bib-0051] McCurry, G. , and D. Pyne . 2022. Planning for managed aquifer recharge projects. Groundwater 60: 583–590. 10.1111/gwat.13226 35811302

[gwat70029-bib-0052] Megdal, S.B. , P. Dillon , and K. Seasholes . 2014. Water banks: Using managed aquifer recharge to meet water policy objectives. Water (Basel) 6: 1500–1514. 10.3390/w6061500

[gwat70029-bib-0053] Meixner, T. , A.H. Manning , D.A. Stonestrom , D.M. Allen , H. Ajami , K.W. Blasch , A.E. Brookfield , C.L. Castro , J.F. Clark , D.J. Gochis , A.L. Flint , K.L. Neff , R. Niraula , M. Rodell , B.R. Scanlon , K. Singha , and M.A. Walvoord . 2016. Implications of projected climate change for groundwater recharge in the western United States. Journal of Hydrology 534: 124–138. 10.1016/j.jhydrol.2015.12.027

[gwat70029-bib-0054] Meles, M.B. , S. Bradford , A. Casillas‐Trasvina , L. Chen , G. Osterman , T. Hatch , H. Ajami , O. Crompton , L. Levers , and I. Kisekka . 2024. Uncovering the gaps in managed aquifer recharge for sustainable groundwater management: A focus on hillslopes and mountains. Journal of Hydrology 639: 131615. 10.1016/j.jhydrol.2024.131615

[gwat70029-bib-0055] Mid‐Kaweah GSA . 2024. 2024 Amended GSP. 2024 Second Amended Groundwater Sustainability Plan. https://www.midkaweah.org/2024‐gsp‐amendment‐public‐review (accessed August 6, 2025).

[gwat70029-bib-0056] Miller, K. , P. Goulden , K. Fritz , M. Kiparsky , J. Tracy , and A. Milman . 2021a. Groundwater recharge to address integrated groundwater and surface waters: The ESPA recharge program, eastern Snake plain, Idaho. Case Studies in the Environment 5: 1223981. 10.1525/cse.2020.1223981

[gwat70029-bib-0057] Miller, K. , A. Milman , and M. Kiparsky . 2021b. Introduction to the special collection. Case Studies in the Environment 5: 1245648. 10.1525/cse.2021.1245648

[gwat70029-bib-0058] Milman, A. , C. Bonnell , R. Maguire , K. Sorensen , and W. Blomquist . 2021a. Groundwater recharge for water security: The Arizona Water Bank, Arizona. Case Studies in the Environment 5: 1113999. 10.1525/cse.2020.1113999

[gwat70029-bib-0059] Milman, A. , K. Bylo , A. Gage , and W. Blomquist . 2021b. Groundwater recharge to support wildlife and water users. Case Studies in the Environment 5: 1235924. 10.1525/cse.2021.1235924

[gwat70029-bib-0060] Nassrullah, H. , S.F. Anis , R. Hashaikeh , and N. Hilal . 2020. Energy for desalination: A state‐of‐the‐art review. Desalination 491: 114569. 10.1016/j.desal.2020.114569

[gwat70029-bib-0061] National Association of Flood and Stormwater Management Agencies . 2006. Guidance for Municipal Stormwater Funding.

[gwat70029-bib-0062] Nylen, N.G. 2021. Surface water quality regulation as a driver for groundwater recharge. Case Studies in the Environment 5: 1124592. 10.1525/cse.2020.1124592

[gwat70029-bib-0063] O'Geen, A.T. , M.B.B. Saal , H.E. Dahlke , D.A. Doll , R.B. Elkins , A. Fulton , G.E. Fogg , T. Harter , J.W. Hopmans , C. Ingels , F.J. Niederholzer , S.S. Solis , P.S. Verdegaal , and M. Walkinshaw . 2015. Soil suitability index identifies potential areas for groundwater banking on agricultural lands. California Agriculture 69: 75–84. 10.3733/ca.v069n02p75

[gwat70029-bib-0064] Olmstead, S.M. , and R.N. Stavins . 2009. Comparing price and nonprice approaches to urban water conservation. Water Resources Research 45. 10.1029/2008WR007227

[gwat70029-bib-0065] Ostrom, E. 2015. Governing the Commons: The Evolution of Institutions for Collective Action, Canto Classics. Cambridge: Cambridge University Press. 10.1017/CBO9781316423936

[gwat70029-bib-0066] Owen, D. 2013. Taking groundwater. Washington University Law Review 91: 253–307.

[gwat70029-bib-0067] Owen, D. 2021. Law, land use, and groundwater recharge. Stanford Law Review 73: 1163–1219.

[gwat70029-bib-0068] Owen, D. 2024. The water district and the state. The Yale Law Journal 134: 1–328.

[gwat70029-bib-0069] Pecharroman, L.C. , C. Williams , N.G. Nylen , and M. Kiparsky . 2021. How can we govern large‐scale green infrastructure for multiple water security benefits? Blue‐Green Systems 3: 62–80. 10.2166/bgs.2021.015

[gwat70029-bib-0070] Peterson, C. , E. Hanak , Z.J. Morales , S. Cole , and K. Greenspan . 2024. Replenishing Groundwater in the San Joaquin Valley: 2024 Update. Public Policy Institute of California 38.

[gwat70029-bib-0071] Ross, A. , and S. Hasnain . 2018. Factors affecting the cost of managed aquifer recharge (MAR) schemes. Sustainable Water Resources Management 4: 179–190. 10.1007/s40899-017-0210-8

[gwat70029-bib-0072] Russo, T.A. , A.T. Fisher , and D.M. Winslow . 2013. Regional and local increases in storm intensity in the San Francisco Bay Area, USA, between 1890 and 2010. Journal of Geophysical Research: Atmospheres 118: 3392–3401. 10.1002/jgrd.50225

[gwat70029-bib-0073] Russo, T.A. , A.T. Fisher , and B.S. Lockwood . 2015. Assessment of managed aquifer recharge site suitability using a GIS and modeling. Ground Water 53: 389–400. 10.1111/gwat.12213 24916466

[gwat70029-bib-0074] San Francisco Water Power Sewer . 2025. Real Estate Services. https://www.sfpuc.gov/construction‐contracts/lands‐rights‐of‐way/real‐estate‐services (accessed March 6, 2025).

[gwat70029-bib-0076] Sax, J.L. 1990. The constitution, property rights and the future of water law. University of Colorado Law Review 61: 257.

[gwat70029-bib-0077] Scanlon, B.R. , S. Fakhreddine , A. Rateb , I. de Graaf , J. Famiglietti , T. Gleeson , R.Q. Grafton , E. Jobbagy , S. Kebede , S.R. Kolusu , L.F. Konikow , D. Long , M. Mekonnen , H.M. Schmied , A. Mukherjee , A. MacDonald , R.C. Reedy , M. Shamsudduha , C.T. Simmons , A. Sun , R.G. Taylor , K.G. Villholth , C.J. Vörösmarty , and C. Zheng . 2023. Global water resources and the role of groundwater in a resilient water future. Nature Reviews Earth & Environment 4: 87–101.

[gwat70029-bib-0078] Searcey, D. , and D. Erdenesanaa . 2023. A Tangle of Rules to Protect America's Water Is Falling Short. *The New York Times* .

[gwat70029-bib-0079] Smith, S.M. , K. Andersson , K.C. Cody , M. Cox , and D. Ficklin . 2017. Responding to a groundwater crisis: The effects of self‐imposed economic incentives. Journal of the Association of Environmental and Resource Economists 4: 985–1023. 10.1086/692610

[gwat70029-bib-0080] Sufyan, M. , G. Martelli , P. Teatini , C. Cherubini , and D. Goi . 2024. Managed aquifer recharge for sustainable groundwater management: New developments, challenges, and future prospects. Water (Basel) 22: 3216.

[gwat70029-bib-0081] Tadych, D.E. , M. Ford , B.G. Colby , and L.E. Condon . 2024. Historical patterns of well drilling and groundwater depth in Arizona considering groundwater regulation and surface water access. JAWRA Journal of the American Water Resources Association 60: 1193–1208. 10.1111/1752-1688.13234

[gwat70029-bib-0082] Tarlock, A.D. 2001. The future of prior appropriation in the New West. Natural Resources Journal 41: 769–793.

[gwat70029-bib-0083] Taylor, G.H. 1961. Recharging Ground‐water reservoirs (Open‐File Report), Open‐File Report. United States Geological Survey.

[gwat70029-bib-0084] Texas House Research Organization . 2000. Managing Groundwater for Texas' Future Growth.

[gwat70029-bib-0085] The Nature Conservancy . 2021. Multi‐Benefit Recharge Project Methodology for Inclusion in Groundwater Sustainability Plans.

[gwat70029-bib-0086] Thompson, B.H. 2000. Tragically difficult: The obstacles to governing the commons. Environmental Law 30: 241–278.

[gwat70029-bib-0087] Thompson, B.H. , J.D. Leshy , R.H. Abrams , and S.B. Zellmer . 2025. Legal Control of Water Resources: Cases and Materials, 7th ed. St. Paul, Minnesota: West Academic Publishing.

[gwat70029-bib-0088] Ulibarri, N. , N. Escobedo Garcia , R.L. Nelson , A.E. Cravens , and R.J. McCarty . 2021. Assessing the feasibility of managed aquifer recharge in California. Water Resources Research 57: e2020WR029292. 10.1029/2020WR029292

[gwat70029-bib-0089] UNESCO . 2021. Managing Aquifer Recharge: A Showcase for Resilience and Sustainability. Paris, France: UNESCO Publishing.

[gwat70029-bib-0090] US EPA . 2013. Enhanced Aquifer Recharge Research. https://www.epa.gov/water‐research/enhanced‐aquifer‐recharge‐research (accessed August 6, 2025).

[gwat70029-bib-0091] US EPA . 2015. Underground Injection Control Well Classes. https://www.epa.gov/uic/underground‐injection‐control‐well‐classes (accessed August 6, 2025).

[gwat70029-bib-0092] Vanderzalm, J. , D. Page , P. Dillon , D. Gonzalez , and C. Petheram . 2022. Assessing the costs of managed aquifer recharge options to support agricultural development. Agricultural Water Management 263: 107437. 10.1016/j.agwat.2021.107437

[gwat70029-bib-0093] de Vries, J.J. , and I. Simmers . 2002. Groundwater recharge: an overview of processes and challenges. Hydrogeology Journal 10: 5–17. 10.1007/s10040-001-0171-7

[gwat70029-bib-0094] Ward, F.A. , and A. Michelsen . 2002. The economic value of water in agriculture: concepts and policy applications. Water Policy 4: 423–446. 10.1016/S1366-7017(02)00039-9

[gwat70029-bib-0095] Yuan, J. , M.I. Van Dyke , and P.M. Huck . 2016. Water reuse through managed aquifer recharge (MAR): assessment of regulations/guidelines and case studies. Water Quality Research Journal 51: 357–376. 10.2166/wqrjc.2016.022

[gwat70029-bib-0096] Zheng, Y. , J. Vanderzalm , N. Hartog , E.F. Escalante , and C. Stefan . 2023. The 21st century water quality challenges for managed aquifer recharge: towards a risk‐based regulatory approach. Hydrogeology Journal 31: 31–34. 10.1007/s10040-022-02543-z 36185762 PMC9512974

